# Adaptive learning in university students’ opinions: Cross-border research

**DOI:** 10.1007/s10639-021-10830-7

**Published:** 2022-01-24

**Authors:** Eugenia Smyrnova-Trybulska, Nataliia Morze, Lilia Varchenko-Trotsenko

**Affiliations:** 1 University of Silesia, Katowice, Poland; 2 Borys Grychenko Kyiv University, Kyiv, Ukraine

**Keywords:** Adaptive learning, Adaptive technologies, LMS Moodle, Students’ expectation, Comparative research

## Abstract

Contemporary education is often based on using e-learning courses, which have become a popular means of delivering didactic material to students. Among the main advantages mentioned is the potential possibility of creating individual ways of learning and teaching. The purpose of this article is to provide a description of the various approaches to adaptive learning, to present the comparative research results of a Polish-Ukrainian study, as well as to highlight the options offered by LMS Moodle (Modular object-oriented dynamic learning environment Learning Management System) for the implementation of adaptive learning and the possibility of taking into account the expectation of students regardless of the country. During the study, an online survey was administered to 59 students at the University of Silesia in Katowice (US), Poland and 121 students at the Borys Grinchenko Kyiv University (BGKU), Ukraine between March–June 2020. All of the students were studying online due to the pandemic caused by COVID-19. The classes were conducted in synchronous and in asynchronous modes using e-learning courses in the Moodle system, as well as MS Teams for Polish students and Google Suite for Ukrainian students. At the same time, the majority of the surveyed students declared that they lack personalization, both in terms of materials and the learning process, which was limited in terms of fulfilment and they would like to have a choice of the level of study.

## Introduction

One of promising educational technologies according to the New Media Consortium (NMC) Horizon Report, 2018 (New Media Consortium (NMC) Horizon Report, 2018) is adaptive learning – the adaptation of content and choice of means for its implementation according to the needs of the educational process which allow participants to increase the effectiveness of the various didactic activities. *Adaptive learning* consists in adapting the learning process to the needs, competences and abilities of the participant of e-training. Artificial intelligence or advanced programming solutions, complex algorithms and extended e-learning platforms play an enormous role in the use of adapting learning to the new e-learning environment.

The *aims of adaptive learning* include firstly, providing appropriate knowledge resources: focus on issues the participant of the educational process/course finds problematic and appropriate revision planning; secondly, eliminating fatigue with the material – the point is to give the participant new, interesting content not studied before; thirdly, fully personalising the learning process.

Adaptive learning is applied, first of all, in the educational process with the use of Information and Communication Technology (ICT) and Artificial Intelligence (AI) supported systems and in training courses for enterprises. Owing to the use of artificial intelligence in business, it is possible to create one extensive training course which will adapt itself to the diverse abilities of employees; namely, the employer can utilise the competences newly acquired by his employees sooner.

The research, conducted by authors from Slovakia, “deals with the principles of e-learning course creation, the design of a specific methodology and personalized e-course model.” (Mudrak et al., 2018, p. 117). They stressed that “to fulfil the conditions of such an e-course, it is important to choose an appropriate LMS on which the authors used the method of SWOT analysis” (p. 117).

The study, elaborated upon by experts from China “presents a new unobtrusive method to model the learners’ personalities in an intelligent Moodle .. using Learning Analytic .. approach with Bayesian network” (Tlili et al., 2019). They emphasize that “to evaluate the accuracy of the proposed approach, an experiment was conducted with one hundred thirty-nine learners in a public university.” (Tlili et al., 2019).

Some researchers have introduced and researched the open-source framework that allows for the generating of adaptive online courses in Moodle. They focused on researching language learning as an example of implementation (Rudian & Pinkwart, 2021). Other authors described the method of achieving the course adaptivity as combined with a personalized approach, as proposed by Limongelli et al. (Shchedrina et al., 2021).

To sum up, adaptive learning offers undoubted *benefits***:** the educational process/e-learning project/training course is fully adapted to the learner, their individual needs, predispositions, capabilities, and the learning process is individualised and personalised on an ongoing basis. There is also a negative aspect: the student is cared for by the implemented elements of artificial intelligence. It is important that this care should not turn into dominance and the student should always be given priority, so that they can make appropriate decisions at all times (Kołodziejczak, n.d.).

The purpose of this article is to provide a description of the various approaches to adaptive learning and personalization within the framework of international background research and literature, to present the comparative research results of a Polish-Ukrainian study, as well as to highlight the options offered by LMS Moodle for the implementation of adaptive learning and the possibility of taking into account the expectation of students regardless of the country.

### The theoretical basics of adaptive learning. Adaptive learning and individualisation and the personalisation of education

*Individualisation* is one of the essential principles of didactics.

Simultaneously, the term *personalization*, refers “to a diverse variety of educational programs, learning experiences, instructional approaches, and academic-support strategies that are intended to address the distinct learning needs, interests, aspirations, or cultural backgrounds of individual students” (https://www.edglossary.org/personalized-learning/).

There are many definitions of *adaptive learning*. One of the descriptions of the term *adaptive learning — or adaptive teaching* — “is the delivery of custom learning experiences that address the unique needs of an individual through just-in-time feedback, pathways, and resources (rather than providing a one-size-fits-all learning experience” (Smart Sparrow Platform, n.d.).

The elements of adaptive learning and teaching were reflected in the works of (Song & Keller, 2001), (Oujezdský et al., 2017; Beckmann et al., 2015; Hill, 2013; Ikwumelu et al., 2015; Kostolányová, 2012) and others. Studies on adaptive learning management systems (Chang et al., 2016; Klubal et al., 2017), and the collaborative design of digital learning (Isaias & Lima, 2018) have shown their effectiveness and the determinants of these methods and forms of learning. Some principles of the creation of study materials for adaptive learning using a ‘virtual teacher’ were studied by researchers in 2014 (Czeczotková & Prextová, 2014), which shows that learning in adaptive forms of education is getting more efficient and rewarding. The research on Adaptive Education Evaluation deals with one of the main issues – the evaluation of the educational process and adaptive textbooks. The authors of this article describe the evaluation conducted after the realization of the analysis of the protocol of students’ opinions regarding the online mode. The research includes the theoretical proposition of the evaluation and its verification in practice (Šarmanová & Kostolányová, 2016). Other authors described the development of the “Tools for Adaptive Learning. Learning Styles” module and the evaluation of the results of its initial testing by PhD students. The module was elaborated upon as part of an open online course co-created by several international research teams within the scope of the IRNet project (“International Research Network for study and development of new tools and methods for advanced pedagogical science in the field of ICT instruments, e-learning and intercultural competences”) (Malach et al., 2017). Some authors explain why adaptive systems are still not used on a large scale, in particular pointing to: inter-operability, open corpus knowledge, usage across a variety of delivery devices, and the design of meta-adaptive systems and the new trends in adaptive educational hypermedia systems research (Somyürek, 2015).

Different authors variously describe and define the concept of personalization in education, which is complex and multi-faceted. The idea of adapting teaching to the student can be attributed to the individualisation of teaching as one of the principles of traditional didactics (Komeński (1956). In the 1950s, the behaviourist concept of learning, so-called ‘programmed learning’ (Skinner, 1968, 1974, 1986) was an attempt to personalize teaching by dividing materials into smaller doses/units and appropriate system responses depending on the results/responses, which would then be broken down or based on an appropriately arranged program containing logically related portions of information on a specific topic.

Later, the computer acting as a “tutor” (Taylor, 1980), or supporting the teaching-learning process, was considered one of the visions of the role of technology in education. The study (2018) defined four key elements that characterize educational delivery in a general way: epistemological character, psychological approach, didactic materialization, technological approach (Bartolomé, Castañeda & Adell, 2018).

In addition, there are several types of knowledge which may affect the individualisation of learning. In the cognitive domain, according to the taxonomy of educational objectives the following kinds of knowledge can be identified (Anderson & Krathwohl, 2001):

- *Factual knowledge –* knowledge of important figures and institutions focusing on adaptive learning, names of companies, knowledge of titles of studies or academic papers.
- *Conceptual knowledge* **–** knowledge of basic definitions, theoretical concepts of adaptive learning/ e-learning, cognitive styles and approaches to learning, the results of particular research studies, models of adaptive (e-)learning.
- *Procedural knowledge –* knowledge of the stages of implementation of adaptive learning, including the evaluation stage, knowledge of the main activities of the teacher/learner in adaptive learning and the processes realised in adaptive e-learning systems. Finally, this area also includes the limits, restrictions, and risks associated with the implementation of adaptive e-learning systems (AEA) and adaptive assessment systems (AAS).
- *Meta-cognitive knowledge –* knowledge of information resources regarding AES/AAS-related products, AES/AAS-related professional networks and communities (Malach et al. 2016).

### Adaptive learning and ICT tools

Experts emphasize that “improving the quality of teaching and learning through the use of new technology is the primary goal of higher education institutions” ... and the introduction of the “ concept of “self-guided studies” in which a student learns to acquire knowledge independently, to develop and apply it.” (Zlatkovic et al., 2020, p. 804). They also stressed that “An adaptive e-learning system is focusing on customizing courses with individual student characteristics.” (Zlatkovic et al., 2020, p. 804).

As a result of the development of ICT, adaptive instruction which takes learners’ unique pre-requisites and needs into account, the theory of adaptive learning is constantly being modified and updated. One of the main pre-requisites of learners is their learning style, which can be categorised according to a number of criteria based on the cognitive and emotional aspects of their personality. The combination of those learning styles in each individual lead to countless real ways of learning, which can be – to a certain degree – affected by current e-learning resources. Those e-learning resources that have predefined features, which allow them to adapt to the learners’ entry characteristics during instruction management or to react to their current results, are AES and/or intelligent AES, both of which are the most important kinds of knowledge for this study.

If the systems are used for the assessment of learning outcomes, they become AAS. With their technical, personnel and research potential, universities may not only make use of both AES and AAS, but also realise their experimental verification and implementation in education (Malach, Kostolányová, Chmura, Nagyová & Prextová, 2016).

The beginning of the twenty-first century marks the beginning of the so-called Intelligent Adaptive Learning (IAL) system, which is aimed at the individualisation and, to a certain degree, personalisation of learning. Intelligent Adaptive Learning is defined as digital learning which is based on students working in modular learning environments where every decision they make is captured and considered within a sound learning theory. Those decisions are then used to guide their learning experiences, to adjust their path and pace within and between classes, and also, to provide their teachers with formative and summative data. By tailoring instruction to each student’s unique needs, current knowledge and interests, this type of instruction ensures that all responses are in compliance with sound pedagogy. The IAL system is designed to: a) be the student’s personal tutor, b) individualise the learning pace, c) regulate the student’s cognitive load, d) adapt the sequence of the curriculum and associated learning experiences and, e) engage students in learning through games (Dreambox Learning, 2011).

The different types of adaptive learning systems were researched and analysed earlier by experts and specialists from various countries. Based on the available research (Fröschl, 2005; Mödritscher, Garcia-Barrios & Gütl, 2004; Karampiperis & Sampson, 2005; Osadcha, Osadchyi, Semerikov, Chemerys & Chorna, 2020), several types of adaptive systems of learning were identified. These are: *Macro-adaptive system; Micro-adaptive system; Aptitude-treatment interactions system (ATI); Intelligent tutoring system (ITS); Adaptive Hypermedia System (AHS); Adaptive Educational Hypermedia System (AEHS); Adaptive Learning Platform (ALP); Deep Learning Platform (ADLP); Computer Adaptive Educational Assessment (CAEA); Learning Objects Difference Engine (LODE). The Micro-adaptive system* (“the system that carries out the adaptation of education at the micro level, is constantly revising and analyzing the profile of pupils on the basis of their activity and provides personified instructions. Such an approach is more effective, as the individual trajectory of learning of every pupil is formed”) is more salient in the context of our research and within the perspective of this could be the *Adaptive Hypermedia System* (AHS) (“the hypermedia system that is built with the help of artificial intelligence and uses the model of a user, in which the pupil’s personal information about knowledge, interests and goals for the adaptation of content and navigation in the hypermedia space is contained”). (Osadcha, Osadchyi, Semerikov, Chemerys & Chorna, 2020, p. 550).

The purpose of creating an adaptive educational system is to fully meet the need for individualization using the technologies of problem-based testing and cognitively interested navigation in the educational material (Pishvanova, 2015).

### The Moodle system in the context of the adaptive learning approach

Researchers from different countries have studied and described their interesting experiences and the practices that led to good results using the Moodle system in conjunction with the *Adaptive Learning Approach*. Some researchers propose an agent-based adaptive architecture to extend Moodle in order to support instructional decisions and adaptive behaviour (Priscila et al., 2009). Providing Adaptivity in Moodle LMS Courses was described by Shchedrina et al. (2021). Researchers also stressed that “the adaptivity of eLearning courses is necessary for the successful implementation of online training in today’s educational system. Prospects for the practical use of the research results lie in the possibility of an international exchange of experience concerning Moodle courses’ adaptivity” (p. 95). Another proposal is “focused on the integration of an adaptive learning architecture into the learning management platform Moodle, application which is conveniently enhanced by the incorporation of mobile devices to the adaptive platform by providing mobile access to Moodle” (Héctor Sánchez, Agudo & Rico (2009). Other experts also “propose an approach that can be integrated with Moodle in order to allow learners to access [to] [a] personalized content according to their level and also allow teachers to effectively control the learning process of their learners. To validate this approach, we propose the SALCM plugin which will be integrated with Moodle as a new module.” (Ezzahraa Louhab, Bahnasse & Talea, 2017, p.1).

An important part of the educational process is feedback, testing and assessment. The example of the LMS Barborka system (Ostrava University) was described in (Prextová, 2016). However, as (Klasnja-Milicevic, Vesin, Ivanovic & Budimac, 2011) emphasises, before we proceed to electronic adaptive testing, let us introduce the basic principles and rules necessary for the proper functioning of adaptive instruction. What does the term ‘adaptive instruction’ actually mean? Paramythis’ description (Paramythis & Loidl-Reisinger, 2003) captures the essence of the adaptive learning environment, which includes four categories: 1) Adaptive Interaction; 2) Adaptive Course Delivery; 3) Content Discovery and Assembly; 4) Adaptive Collaboration Support.

The fourth and final category captures communication between people (so-called ‘social interaction’) and collaboration toward common goals. It is important to support communication, collaboration, and cooperation as the individualist approach to learning can lead to complete isolation.

A different view is offered by (Spencer, 2011), who says that there are 4 stages of personalization in teaching. According to Spencer (2011), the elements of adaptive learning are): 1) Standardization; 2) Differentiation; 3) Adaptation; 4) Personalization. All the levels can be taken as the gradual development of the teacher, their ability to improve their material-preparation skills and their skills regarding the organization of the education process. In (Hill, 2013), the author also distinguishes between the various types of teaching that deal with the adaptive learning process and points to some differences between the concepts of adaptivity and personalization, which are: 1) Differentiated learning; 2) Personalized learning; 3) Adaptive learning.

Adaptive learning takes into account the student’s results during the entire time of instruction. It is a dynamic process as the student’s “path” can be changed all the time (Prextová, 2016). For a long time, the theory of adaptive education (TAE) has been the research focus of a team of experts at the Department of ICT of the University of Ostrava, which mirrors the research results – system development (Drápela, 2014); system fine-tuning using simulation (Kostolányová, 2013); and the proposal, creation, and implementation of rules (Takács, 2014). The theory is being further developed as new aspects are constantly emerging. From what researchers know, they assume that if the TAE is applied predominantly in the first two stages, then in the remaining two stages, adaptive testing can be applied (Prextová, 2016). Later in the course, testing in adaptive LMS can also be conducted.

### Learning styles and multiple intelligences in the context of the adaptive learning approach

Learning styles refer to a range of competing and highly contested theories that aim to account for differences in individuals’ learning styles. The many theories share the proposition that humans can be classified according to their ‘style’ of learning but differ in how the proposed styles should be defined, categorised and assessed. A common concept is that individuals differ in how they learn. Based on an analysis of the research results, publications and the opinions of scientists, it can be concluded that in the previous century, two main important theories were forwarded in an attempt to interpret human differences and to design pedagogical and psychological models for learning effectiveness around these differences. Learning-style theory has its roots in the psychoanalytic community, research was conducted most importantly by (Jung, 1927; Briggs and Myers, 1977; Butler, 1984; Gregorc 1985; McCarthy, 1982; Kolb, 1984; Fleming, 2001; Silver & Hanson, 1995). On the other hand, the study conducted by (Silver, Strong & Perini, 1997) explains that the multiple intelligences theory is the fruit of cognitive science and reflects an effort to rethink the theory of measurable intelligence embodied in intelligence testing, which has been analysed in-depth by (Silver and Strong, 1997; Gardner, 1993; Amthauer, 1970; Raven, 1936).

In particular, according to the model developed by (Fleming, 2001), that is based on his experiential learning model, the following approaches must be incorporated: Accommodator; Converger; Diverger; Assimilator. Another theory was also put forward by David Kolb, well known for his Kolb Cycle or a model of adults “effective learning”. Incidentally, the Kolb Cycle also verges on the reputation of a scientific myth, as it does not describe “the best” or “universal” way of learning, regardless of whether they are adult or young learners. In Kolb’s concept, each style is matched with a particular stage of his cycle: Converging style (preferred by “empiricists” – persons who appear to learn best by experiencing), Assimilating style (preferred by “analysts” – persons who learn by gathering and analysing information), Diverging style (preferred by “theoreticians” – persons who learn by analysing relationships in theoretical models), − Accommodating style (preferred by “pragmatists” – persons who learn by applying new knowledge in practice).

Barbe et al. proposed the concept of VAK and identified the acronym as: Visualising modality; Auditory modality; Kinaesthetic modality (Barbe, Swassing & Milone, 1979).

The VARK concept (Silver & Hanson, 1995; Fleming & Mills, 1992; Fleming & Baume, 2006) Fleming, (n.d.), adds a “verbal” style to the visual, aural/auditory and kinaesthetic styles, in which the most important role is played by reading and writing (it is clear that in this division “visual” learners no longer learn better due to reading; the written text is not considered as a visual but a verbal stimulus). We can also notice these preferences: reading/writing and multimodal.

Some researchers proposed a concept of learning styles consisting of as many as seven styles, as well as categorized abilities and sample vocations for the seven intelligences, by learning style (Silver, Strong & Perini, 1997): verbal (linguistic), logical (mathematical), visual (spatial), physical (bodily-kinaesthetic), aural (aural-musical), social (interpersonal), solitary (intrapersonal) (Goetz, n.d.).

As one may conclude, both multiple intelligences and learning styles can work together and provide the researchers, teachers and learners with good tools for enhancing the effectiveness of the education process (Silver, Strong & Perini, 1997).

Simultaneously, the research around „learning styles” are being eagerly discussed at present. Currently, past popular styles are sometimes debunked or critically assessed by different experts (e.g. Willingham, Hughes & Dobolyi, 2015). Therefore, because learning styles taking the above ideas into account need a balanced approach.

### Learning styles (VARK) and the designing of lessons in Moodle LMS

As experts stressed in order “to increase the effectiveness and efficiency of the e-learning system, it is necessary first of all to consider the characteristics of students and their learning styles” (Zlatkovic et al., 2020, p. 803).

**Fig. 1:**
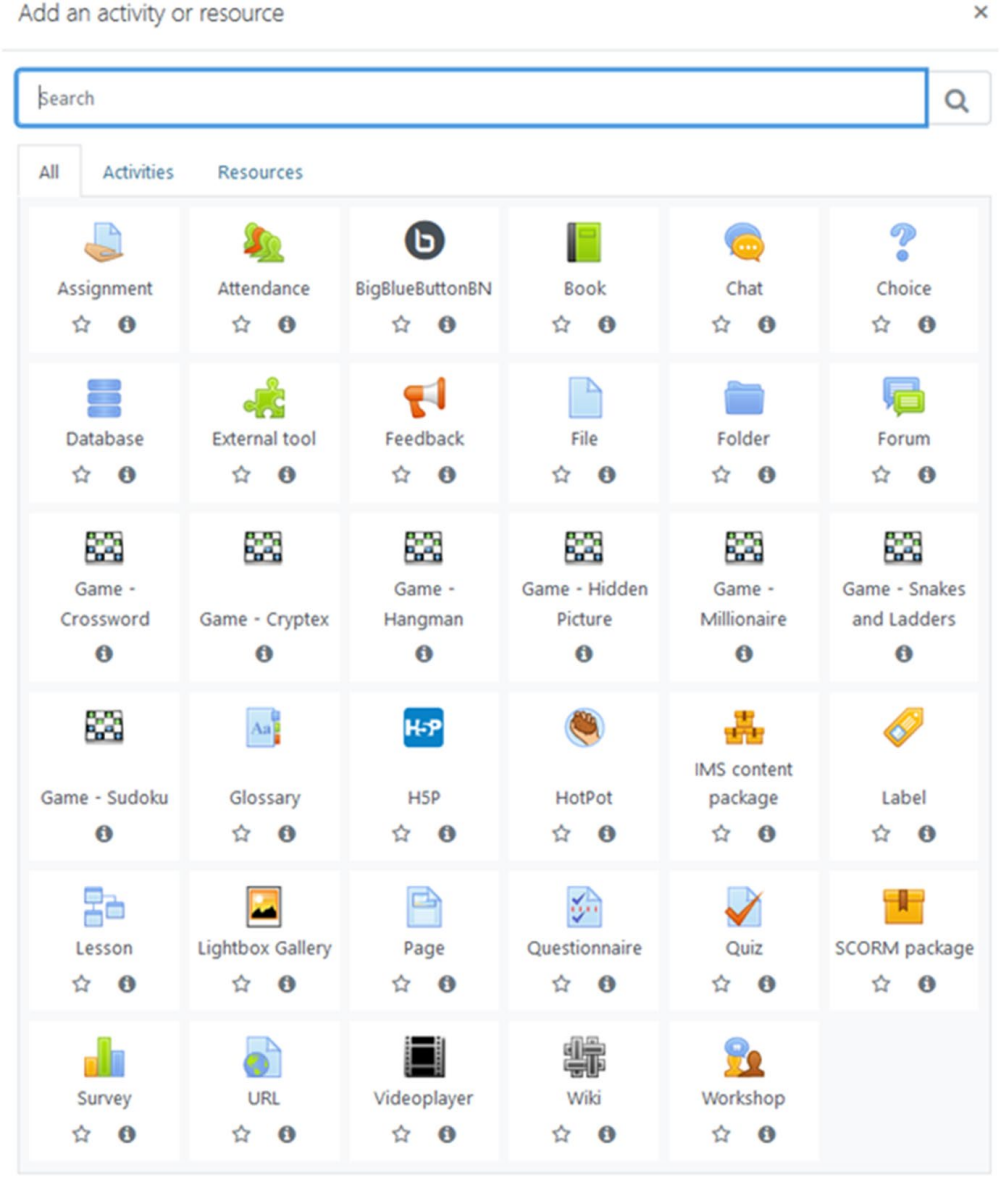
LMS Moodle 3.8.1 activities

The new version Moodle (Modular Object Oriented Dynamic Learning Environment) 3.8.1 LMS (Learning Management System) (Moodle LMS. New features, n.d.) offers a variety of different activities and new options (Fig. 1). Full details of the release with updated technical information is available in the Moodle 3.8 release notes (Moodle 3.8. Release notes, n.d.). The main Moodle activities available and permanently updated include among others, e.g.: Assignment, Book, Chart, Choice, File, Folder, Forum, Game, Glossary, Hotpot, label, Lesson, Page, Questionnaire, Quiz, Survey, URL (Uniform Resource Locator), Wiki, Videoplayer, Workshop.

Among the new activities available in Moodle 3.8.1 LMS version is the H5P (HTML5 (HyperText Markup Language 5) Package) interactive content (which engages with interactive content in Pages, Labels, Books and more). In order for teachers to upload and display the existing H5P files in their courses, the appropriate H5P content types and libraries must be available on the site (Moodle 3.8 Release notes H5P, n.d.).

**Fig. 2:**
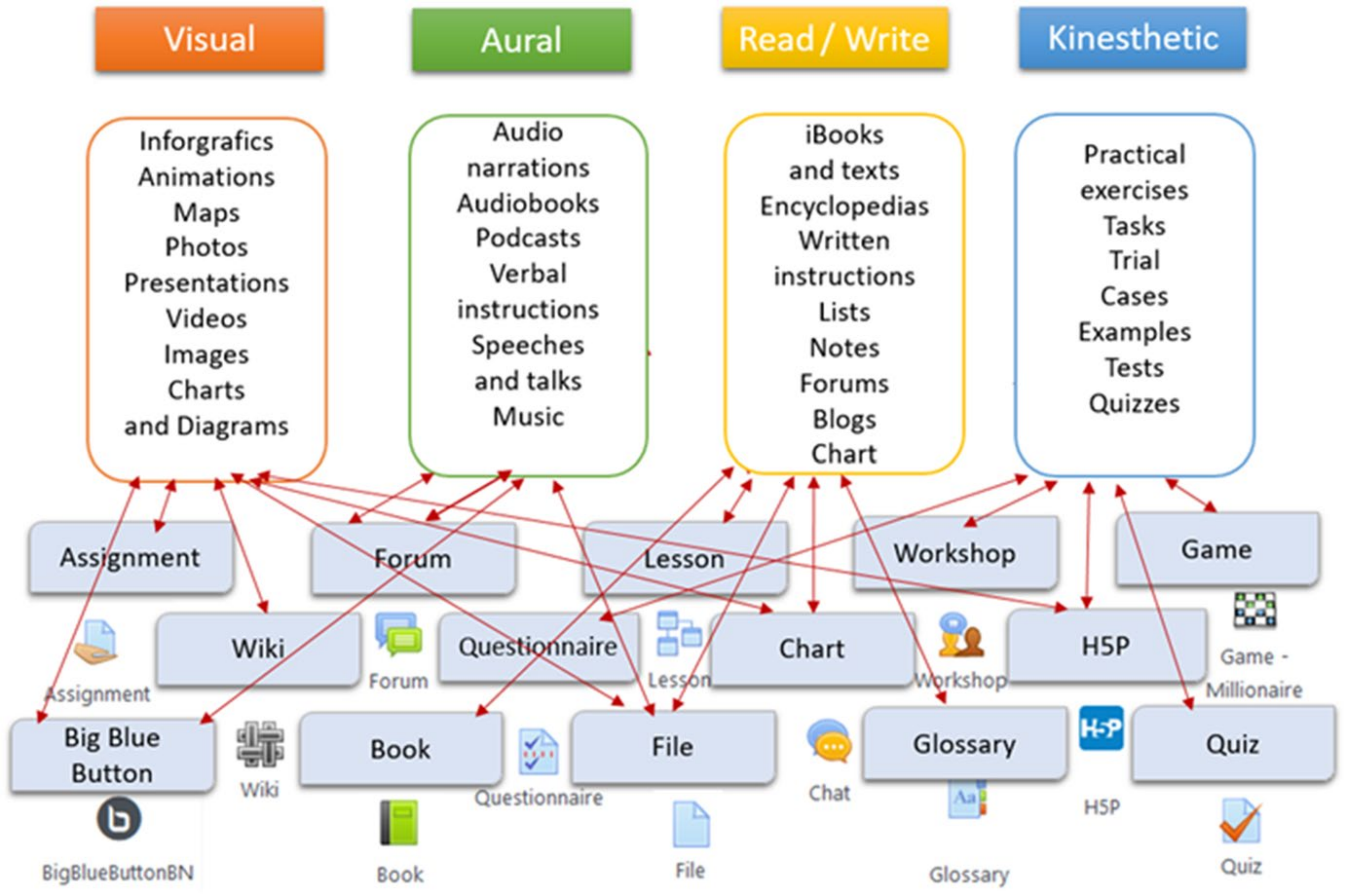
Utilization of Moodle activities for the preparation of learning materials using the VARK model

The Moodle LMS activities mode allows for the simultaneous utilization of different types of materials to provide better information acquisition by students with different learning styles (VARK Fleming, n.d.) (Fig. 2). As stressed above, the VARK (Visual, Aural/Auditory, Reading, Kinaesthetic Styles concept proposed and identified by the acronym as: Visualising modality; Auditory modality; Kinaesthetic modality and Verbal (Read / Write) style. In the Moodle system, teachers can prepare and use different types of content depending on, e.g. a VAKR defined style and thus, suggested for students with adequate preferences and an individual learning approach. In particular, Infographics, Animations, Maps, Photos, Presentations, Videos, Images for the *Visual* mode; Audio narrations, Audiobooks, Podcasts, Verbal instructions could be used for the *Aural* mode; iBooks, and texts, Encyclopedias, Forums, Blogs could be used for the *Verbal* (Read / Write) mode and Practical exercises; Tasks, Examples, Tests, Quizzes could be used for the *Kinaesthetic* mode. As shown in Fig. 2 all these activities are successfully supported via Moodle system components.

**Fig. 3:**
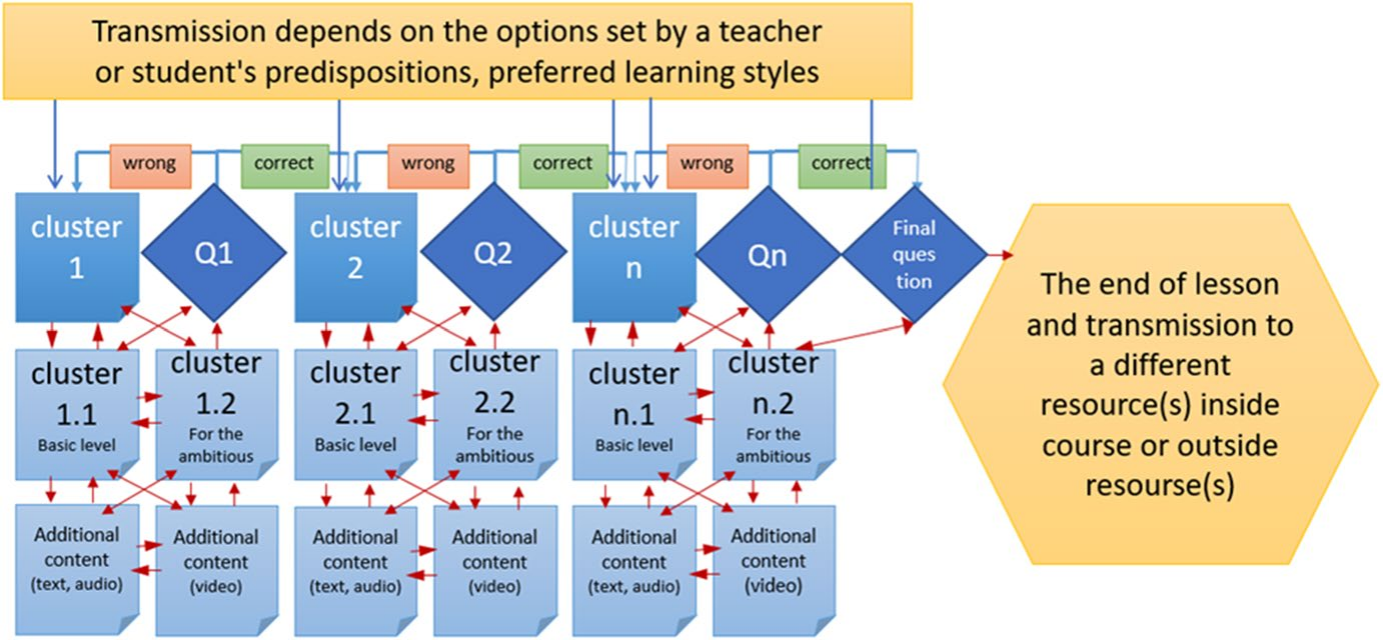
Proposal of a lesson structure with adaptive components

Typically, a teacher can use the Lesson activity mode to provide consequent theoretical materials (meant as a set of clusters with didactic materials) or to organize learning activities where different individual trajectories of a lesson are offered using transactions between clusters, adding extra clusters and pages with questions (true /false, multichoice, matching, short answer questions, etc.) (Morze, Varchenko-Trotsenko, Terletska & Smyrnova-Trybulska, 2021) (Fig. 3).

Depending on the student’s answers and the way a teacher uses the Lesson activity mode, a student can either go to the next cluster or return to the previous cluster or be directed in a different way that corresponds to a particular student’s needs. If it is required, a Lesson can be assessed and/or designed for different difficulty levels, and can be a part of an adaptive assessment. The type of the lesson can be chosen by the lecturer depending on the students’ educational needs and the way it will be used - for support of in-class activities online or for self-study offline. (Morze, Varchenko-Trotsenko, Terletska & Smyrnova-Trybulska, 2021).

One of the tools that can be helpful for the implementation of adaptive materials, in particular regarding assessment is a Quiz, as some options in the quiz resource allow students not only to answer questions, but also to interact with the system, and impact possible frameworks of getting a grade.

The design of such questions is provided with the help of the option Question behaviour that is used to choose is the test passing mode by students. The details of the quiz modes were described in Morze, Varchenko-Trotsenko, Terletska & Smyrnova-Trybulska (2021).

## Materials and methods

### Materials

The research was conducted among students of 1st (B.A.) and 2nd (M.A.) degree courses in the framework of different subjects at different faculties at the University of Silesia in Katowice (UŚ) and at the Borys Grinchenko Kyiv University (BGKU) during March–June 2020 online due to the pandemic caused by COVID-19. The classes were conducted in synchronous and in asynchronous mode using e-learning courses in the Moodle system, as well as MS Teams for Polish students and Google Suite for Ukrainian students. During the study, an online survey was distributed to 59 students at the UŚ and 121 students at the BGKU. The Questionnaire was prepared and developed in electronic mode and shared online in Google Forms for Polish students and for Ukrainian students. The draft results were partly described in Morze et al. (2021).

As we can see, both the University of Silesia and BGKU have extensive experience in implementing e-learning in the educational process to support various disciplines and create an e-environment. At the same time, it was worth examining how students evaluate e-learning courses and what their expectations, proposals and suggestions for their improvement are.

The University of Silesia e-learning platforms provide students with more than 8000 h of effective work (Distance learning platforms, n.d.). The Distance Learning Centre of the University of Silesia (n.d) provides technical support, course administration and training for teaching staff and students (Distance Learning Centre of the University of Silesia, n.d.). On the platforms of the Distance Learning Centre at the University of Silesia, since the beginning of the activity, there have been 133,850 registered users who used the teaching support platform in the remote mode. In the last year, 24,800 active platform users have been registered. The majority of academic teachers successfully participated in teacher training courses in personal or online mode (20 h total) and now hold certificates as tutors and as e-learning course authors in the use of the Moodle system. Many courses/modules are already supported by the Moodle system – a total of 1556 e-learning teaching support courses have been developed.

In particular, the distance learning platform of the Faculty of Arts and Sciences of Education (WSNE), University of Silesia based on the Moodle LMS system serves various purposes, including, but not limited to:

- providing support for teaching programme courses/modules, conducted in the full-time and part-time mode (hybrid learning), being fully online during the Covid-19 pandemic;
- preparing pre-service teachers for learning in person and in remote mode – to use e-learning in their own profession and to perform the role of a tutor,
- providing assistance with scientific research and pedagogical experiments carried out by academic staff.

Additionally, students can use the following system sources and services such as:

- projects and Inter-departmental platforms, e.g. (UPGOW Project platform, n.d.).
- Repository of the University of Silesia (RE-BUŚ) was established in order to disseminate the scientific achievements of employees, promote scientific research conducted at the University of Silesia, and support didactic processes. RE-BUŚ contains full texts of the publications of employees, associates, doctoral students and students of the University of Silesia (Repository  of  the University of Silesia (RE-BUŚ), n.d.) .
- Scientific Information Centre and Academic Library (CINiBA) sources - joint project of the University of Silesia and the University of Economics in Katowice, a hybrid library – free access to books, magazines, databases, electronic texts, audio-visual materials, multimedia (Scientific Information Centre and Academic Library (CINiBA), n.d.).
- TV - UŚ broadcasts and publishes a range of materials covering university news and events, promotion and information, current issues and campaigns. TV UŚ and DLC UŚ also run online broadcasts of online classes and lectures on YouTube Channel (TV UŚ YouTube Channel, n.d).
- The MOOCs available on the first Polish MOOCs platform www.Navoica.pl and other Internet sources

The e-learning development of the University of Silesia was one of the main goals of the Strategy of the development of the University in 2012–2020 as well as in 2020–2025 (Strategy development of the University of Silesia in 2012-2020 (n.d.); Strategy development of the University of Silesia in 2020-2025. (n.d.)). and because of the global transition to online learning during the pandemic and the introduction of the lockdown did not cause any special problems due to the fact that the university was quite well prepared and had implemented e-learning since 2005.

The Borys Grinchenko Kyiv University, Ukraine (BGKU) also actively provides e-learning to the educational process and dynamically develops the e-environment for supporting a comfortable and effective online learning experience. In particular, at the BGKU:

- the Computer Science Laboratory is run for coordinating and supporting the implementation of e-learning platforms. In accordance with the Strategy (Program) of development of the Borys Grinchenko Kyiv University for 2018–2022, the roadmap of the quality of the educational process introduced the use of electronic individual plan of the teacher as part of the digital personal office (Strategy (program) of the Borys Grinchenko Kyiv University development for 2018-2022, n.d.).
- More than 2601 e-learning courses were conducted, 336 e-learning courses were certificated according to a special procedure based on a long time reviewing process by experts. The e-learning platform is available at (The e-learning platform of Borys Grinchenko Kyiv University, n.d.).

The E-environment includes: E-portfolio; E-learning; Institutional Electronic repository; repository; Electronic catalogue; WIKI; Electronic publications; the basis graduation works (BA, MA, PhD Theses); Scientific conferences and seminar proceedings, Microsoft Cloud Services; Authorized CERTIPORT certification centre; Cisco Electronic testing and other components (E-environment of Borys Grinchenko Kyiv University, n.d.).

- The International legal framework and basis of the e-environment development: UNESCO Recommendations, European Quality Standards for Higher Education, European ICT Competence Framework 2.0, Law on Higher Education of Ukraine, investigated the effects of macro-trends. As a result, the informational and educational e-environment of the Borys Grinchenko Kyiv University was created (Morze & Buynytska, 2015). Necessary conditions for the development of a quality e-environment of the university was the availability of developed and approved corporate standards, as well as the development of indicators and indicators to ensure internal quality standards of educational activities. The strategy (program) of the University development for 2018–2022 is available at (Strategy (program) of the Borys Grinchenko Kyiv University, n.d.).

As we can see, both the University of Silesia and BGKU have extensive experience in implementing e-learning in the educational process to support various disciplines and create an e-environment. At the same time, it was worth examining how students evaluate e-learning courses and what their expectations, proposals and suggestions for their improvement were.

The Research Questions, based on the analysis of the background research, as well as the experience of the authors, were developed at the beginning of the current research project, and were specified as follows:

- RQ1: What digital educational tools do students from different countries prefer?
- RQ2: What positive features and conditions for using e-learning courses do students from different countries point to?
- RQ3: What shortcomings in using e-learning courses do students from different countries point to?
- RQ4: What activities can improve the quality of e-learning courses, in particular within the context of the adaptive learning features and taking into account the individual expectation and preferences according to students from different countries?
- RQ5: How do students from different countries rate the usefulness and effectiveness of ICT-tools and Internet services, in particular within the context of the adaptive learning features and taking into account their individual expectations and preferences during their distance learning classes during the Covid-19 (is an acronym that stands for coronavirus disease of 2019) pandemic on a scale of 1–5?

### Methods

The study analysed the parameters that were collected by means of qualitative data on an ordinal scale or a nominal scale. The variables on the ordinal scale are represented by the median value and their minimum and maximum value. For the qualitative data, these were plotted on an ordinal and nominal scale, for each, the number of the examined feature in the analysed part is also given, along with the material as a percentage. The selected variables are also presented in the table’s summary. Variables on the nominal scale (dimension tables 2 × 2) were analysed using the Chi-square test of independence or the exact Fisher and Mann-Whitney test.

The analysis was performed using two-sided statistical tests at the significance level of α = 0.05 in the Dell Inc. statistical package. (2016). Dell Statistica (data analysis software system), version 13. (www.software.dell.com).

## Results

In the next part of the article, an analysis of the comparative research results will be presented. These results were evaluated by students from both Poland and the Ukraine and were collated using Google Forms. Appendix 1. contains the questions of the research survey.

The following Tables show the statistically analysed results, followed by a few comments on the various aspects of each survey question.

**Fig. 4:**
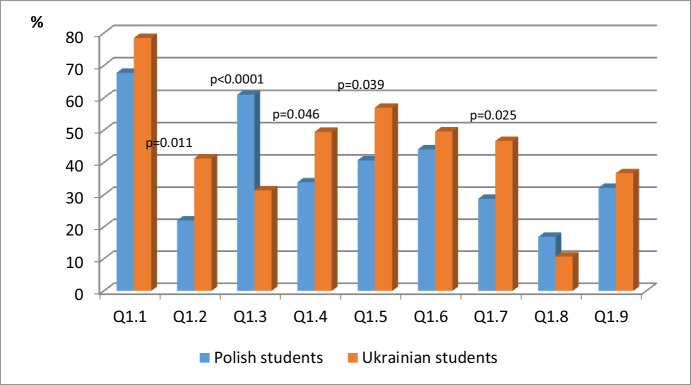
The statistical analysis of research question Q1: What digital educational tools do you prefer?

Table 1, as well as Fig. 4, includes the statistical analysis of data concerned with Q1. *What digital educational tools do you prefer? As well as* Fig. 4*shows.*

**Table 1:** The statistical analysis of research question Q1: What digital educational tools do you prefer?

	Polish students	Ukrainian students	
	n (%)	n (%)	p
Q1.1	40 (67.8)	95 (78.5)	.119^a^
Q1.2	**13 (22.0)**	**50 (41.3)**	**.011**^**a**^
Q1.3	**36 (61.0)**	**38 (31.4)**	**.000**^**a**^
Q1.4	**20 (33.9)**	**60 (49.5)**	**.046**^**a**^
Q1.5	**24 (40.7)**	**69 (57.0)**	**.039**^**a**^
Q1.6	26 (44.1)	60 (49.6)	.487^a^
Q1.7	**17 (28.8)**	**56 (46.7)**	**.025**^**a**^
Q1.8	10 (16.9)	13 (10.7)	.242^a^
Q1.9	19 (32.2)	44 (36.7)	.583^a^

^a^Chi squared test

The chi-square p = .119 test showed no significant relationship between nationality and electronic training. Q1.1 shows no significant moderation by nationality, but does show that 78.5% of Ukrainian students and 67.8% of Polish students showed their preference for electronic training (e-learning courses) and this is interesting, as it is the main topic of this study. The students from both countries gave a positive assessment of their participation in e-learning courses, which are considered to be their preferred educational tools. The reason and the determining factor may be the ease of access to didactic materials (24 * 7 * 356), their variety and the differentiated nature of the content; which supports all stages of the educational process; self-control; working in asynchronous and synchronous modes; sending tasks to the instructor, receiving feedback, among other aspects.

The chi-square p = .011 test showed a significant association between nationality, and 2 (Massive Open Online Courses (MOOCs)). 78.5% of Polish students chose 0. and in the group of Ukrainian students, 59%. So, students from the Ukraine significantly more often chose massive open online courses (MOOCs). The chi-square p = .000 test found a significant relationship between nationality, and 3 (Personalized study materials). While Polish students chose 1 61.0% of the time, only 31.4% of Ukrainian students chose the same answer. So, Polish students significantly more often chose personalised study materials. The chi-square p = .046 test found a significant association between nationality, and 4 (Video channel). For this question, 33.9% of Polish students 33.9% chose 1, compared with 49.5% of Ukrainian students. Therefore, Polish students significantly more often chose video channels. The chi-square p = .039 test found a significant association between nationality, and 5 (Mobile applications). The percentages for this part are noteworthy with 40.7% of Polish students choosing 1, as compared to 57.0% of Ukrainian students. So, Ukrainian students significantly more often chose applications.

The chi-square p =. 487 test did not show any significant association between nationality and 6 (Social networks).

The chi-square p = .025 test found a significant association between nationality, and 7 (Search engines for the necessary materials). 28.8% of Polish students chose 1, while 46.7% of Ukrainian students chose the same answer; therefore, students from the Ukraine significantly more often chose search engines.

The chi-square p = .242 test found no significant association between nationality and 8 (audio books). The chi-square test p = .583 found no significant association between nationality, and 9 (e-books).

**Fig. 5:**
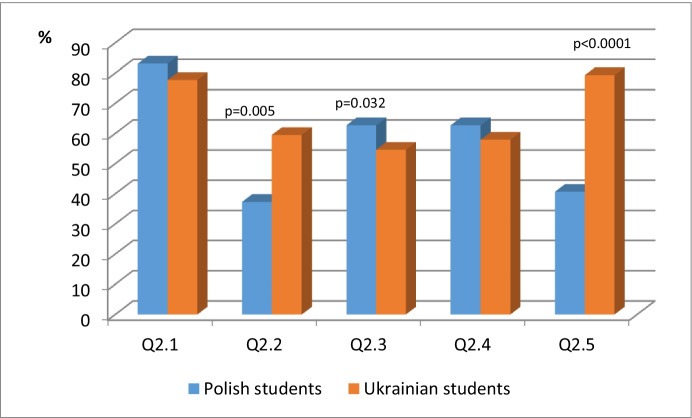
The statistical analysis data concerned with Q2: The University uses the e-learning system, please indicate the positive features and conditions for using e-learning courses

Table 2, as well as Fig. 5, includes the statistical analysis of data concerned with Q2: *The University uses the e-learning system, please indicate the positive features and conditions for using e-learning courses.*

**Table 2:** The statistical analysis data concerned with Q2: The University uses the e-learning system, please indicate the positive features and conditions for using e-learning courses

	Polish students	Ukrainian students	
	n (%)	n (%)	p
Q2.1	49 (83.1)	94 (77.7)	0.403^a^
**Q2.2**	**22 (37.3)**	**72 (59.5)**	**0.005**^**a**^
**Q2.3**	**42 (71.2)**	**66 (54.6)**	**0.032**^**a**^
Q2.4	37 (62.7)	70 (57.9)	0.533^a^
**Q2.5**	**24 (40.7)**	**96 (79.3)**	**0.000**^**a**^

^a^Chi squared test

The chi-square p = .403 test showed *no significant* association between nationality and 1 (Possibility of convenient, comfortable time for study). The chi-square p = .533 test showed no significant association between nationality and 4 (Availability of materials in various formats (texts of lectures, presentations, text files, videos, discussion forums, tests, etc.).

The chi-square p = .005 test showed a significant association between nationality, and 2 (Possibility of completing the course tasks and sending them to be checked). The percentages were recorded as follows, 37.3% of Polish students chose 1, as compared to the group of surveyed Ukrainian students - 59.5%, so students from the Ukraine significantly more often have the opportunity to perform tasks. The chi-square p = .032 test found a significant association between nationality, and 3 (Possibility of returning to course materials and individual topics several times). The following percentages were recorded, 71.2% of Polish students chose 1, compared to 54.6% of Ukrainian students, so students from Poland significantly more often have the possibility of returning several times to the material and topics.

The chi-square p < 0.000 test showed a significant association between nationality and 5 (Ability to perform online/offline tasks and send the results of their implementation to the course instructor).

**Fig. 6:**
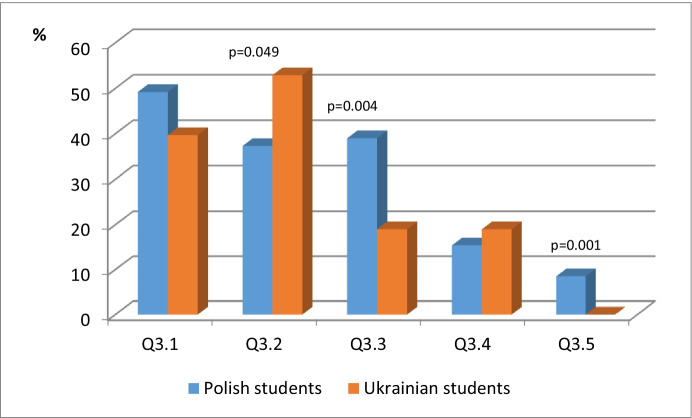
Statistical analysis of data concerned with Q3. Please indicate any shortcomings in the use of e-learning courses

The results concerning 5 showed that 40.7% of Polish students chose 1, compared to 79.3% of Ukrainian students, so students from the Ukraine significantly more often have the opportunity to perform tasks in a timely manner. Table 3 as well as Fig. 6 includes the statistical analysis data concerning Q3. *Please indicate any shortcomings in the use of e-learning courses.*

**Table 3:** Statistical analysis of data concerned with Q3. Please indicate any shortcomings in the use of e-learning courses

	Polish students	Ukrainian students	
	n (%)	n (%)	p
Q3.1	29 (49.2)	48 (39.7)	0.227^a^
**Q3.2**	**22 (37.3)**	**64 (52.9)**	**0.049**^**a**^
**Q3.3**	**23 (39.0)**	**23 (19.0)**	**0.004**^**a**^
Q3.4	9 (15.4)	23 (19.0)	0.536^a^
Q3.5	5 (8.5)	0 (0.0)	0.001^a^

^a^Chi squared test

The chi-square p = .227 test showed no significant association between nationality and 1 (Teaching materials do not take into account the specificity of perception of information by students). The chi-square p = .536 test showed no significant relationship between nationality, and 4 (Test homogeneity).

The chi-square p = .049 test showed a significant association between nationality, and 2 (Limited training time within the course). We see the percentages, i. e. in the group of Polish students, 37.3% chose 1, compared to 52.9% of Ukrainian students, so students from Ukraine more often have significantly limited time.

The chi-square p = .004 test showed a significant association between nationality, and 3 (Lack of personalization in the learning process). We see the percentages, i. e. in the group of Polish students 39.0% choose 1, and in the group of Ukrainian students 19.0%, so Polish students significantly more often lack personalization.

The chi-square p = .001 test showed a significant association between nationality, and 5 (Other). In the group of Polish students, 8.5% chose 1, and in the group of Ukrainian students 0%.

Among the answers *Other* there were: More tasks involving the participant would be great; I do not have any opinions; Lack of clarification in commands; Organizing activities at convenient times; I have no objections to the class form, the only minus is not very specific tasks.

The Polish students – respondents from the University of Silesia represented different Faculties and specialities, e. g. Faculty of Arts and Sciences of Education; Faculty of Societal Sciences; Faculty of Law and Administration. Students were studying in the first semester of the Bachelor’s or MA Programme.

The Ukrainian students – respondents from the Borys Grinchenko Kyiv University also represented different Faculties and specialities, e. g. Faculty of Information Technology and Management, Pedagogical Institute, Institute of Journalism, Institute of Philology. Students were studying in the first semester of a Bachelor’s or MA Programme.

**Fig. 7:**
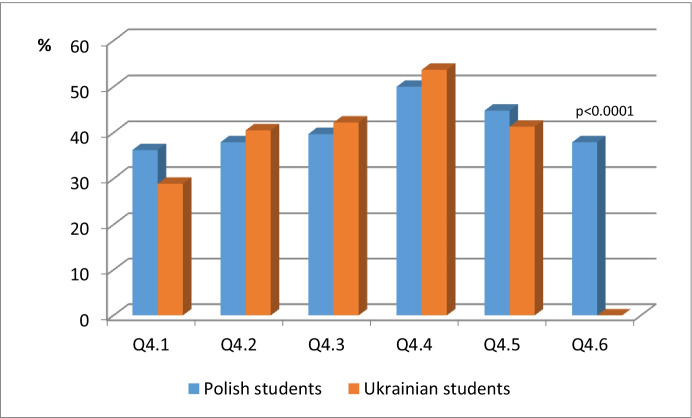
The statistical analysis of data concerned with Q4: Please indicate what activities can improve the quality of e-learning courses

Table 4, as well as Fig. 7, includes the statistical analysis of data concerned with Q4: *Please indicate what activities can improve the quality of e-learning courses.*

**Table 4:** The statistical analysis of data concerned with Q4: Please indicate what activities can improve the quality of e-learning courses

	Polish students	Ukrainian students	
	n (%)	n (%)	p
**Q4.1**	**21 (36.2)**	**35 (28.9)**	**0.325**^**a**^
**Q4.2**	**22 (37.9)**	**49 (40.5)**	**0.743**^**a**^
**Q4.3**	**23 (39.7)**	**51 (42.2)**	**0.751**^**a**^
**Q4.4**	**29 (50.0)**	**65 (53.7)**	**0.641**^**a**^
**Q4.5**	**26 (44.8)**	**50 (41.3)**	**0.657**^**a**^
Q4.6	22 (37.9)	0 (0.0)	<0.0001^b^

^a^Chi squared test

^b^Fisher exact test

The chi-square p = .325 test showed no significant association between nationality and 1 (Choosing the order of studying material and performing tasks).

The chi-square p = .743 test showed no significant association between nationality and 2 (Determining the scope of differentiating knowledge provided by the course (basic, standard, advanced).). The chi-square p = .751 test did not show any significant association between nationality, and 3 (Selection of learning dates from the different topics of the training materials). The chi-square p = .641 test did not show any significant association between nationality and 4 (Choosing the format for the delivery/presentation of educational materials). The chi - square p = .657 test did not reveal any significant association between nationality and 5 (Choosing the level of complexity of tasks and tests). Conclusions from the test did not show any significant relationship between nationality and choosing answer options 1, 2, 3, 4 and 5. Regardless of their nationality, students have the same suggestions and expectations and point to the same actions that can improve the quality of e-learning courses.

Fisher’s exact test <= 0.000 found a significant association between nationality and Other. We read the percentages, for example, in the group of Polish students 37.9% choose 1, and in the group of Ukrainian students 0%, so Polish students significantly more often chose Other.

Additional Q5. Comments and suggestions for improving the functionality of e-learning courses during the Covid-19 quarantine pandemic.

Q5. Please rate the usefulness and effectiveness of tools and websites during the distance learning requirement during the Covid-19 quarantine pandemic on a scale of 1–5 (1 = the lowest grade, 5 = the highest grade):

1. Electronic training (e-learning courses)
2. Massive Open Online Courses (MOOCs)
3. Personalized study materials
4. Video channels
5. Mobile applications
6. Social media
7. Required material search engines
8. Audio books (audiobooks)
9. E-books (ibooks)
10. Other

**Fig. 8:**
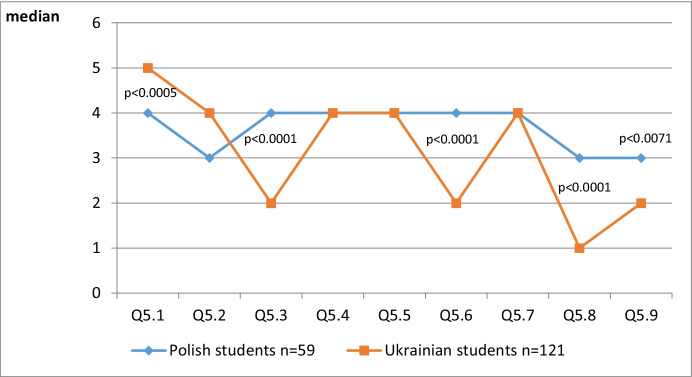
Descriptive statistics and Mann-Whitney test statistics

Table 5, as well as Fig. 8, includes the Descriptive Statistics and Mann-Whitney test Statistics.

**Table 5:** Descriptive statistics and Mann-Whitney test statistics

	Polish students n = 59	Ukrainian students n = 121	
	me (min – max)	me (min – max)	p-volumes^a^
Q5.1	4 (1–5)	5 (1–5)	**0,0005**
Q5.2	3 (1–5)	4 (1–5)	0,8957
Q5.3	4 (1–5)	2 (1–5)	**0,0000**
Q5.4	4 (1–5)	4 (1–5)	0,7123
Q5.5	4 (1–5)	4 (1–5)	0,2022
Q5.6	4 (1–5)	2 (1–5)	**0,0000**
Q5.7	4 (1–5)	4 (1–5)	0,7214
Q5.8	3 (1–5)	1 (1–5)	**0,0000**
Q5.9	3 (1–5)	2 (1–5)	**0,0071**

The Mann-Whitney test found significant differences in questions 1, 3, 6, 8 and 9, with ‘p’ values ​​lower than the significance level α = 0.05. In question 5 “Please rate the usefulness and effectiveness of tools and websites during the distance learning requirement of the Covid-19 quarantine pandemic on a scale of 1-5 (1 = the lowest grade, 5 = the highest grade): Ukrainian students, on average, chose and rated the variant 1 “Electronic training (e-learning courses)“ out of 5, and Polish students - 4. In question 5, Ukrainian students, on average, chose and rated option 2 “Massive Open Online Courses (MOOCs)“ at 4, and Polish students - 3. In question 5, Ukrainian students, on average, selected and rated option 3 “Personalized study materials“ at 2, and Polish students - 4. In question 5, Ukrainian students, on average, chose and rated option 4 “Video channels“ at 4, Polish students also at 4. In question 5, Ukrainian students, on average, chose and rated option 5 “Mobile applications“ at 4, Polish students also at 4. In question 5, Ukrainian students, on average, chose and rated option 6 “Social media“ at 2, and Polish students - 4. In question 5, Ukrainian students chose and rated option 7 “Required material search engines“ at 4, and Polish students - 4. In question 5, Ukrainian students, on average, chose and rated option 8 “Audio books“ at 1, and Polish students - 3. In question 9, Ukrainian students chose and rated option 9 “E-books” at 2, and Polish students - 3.

We can see that there was a volume median of 4.0 (Polish students) for answers: electronic training (e-learning courses); personalised study materials; video channels; mobile applications; social media; required material search engines, and 5 (Ukrainian students) for answers electronic training (e-learning courses) and 4 for MOOCs, video channel, mobile applications, required material search engines. This means that it is these IT-tools and Internet services that students consider to be most effective during the time of distance learning resulting from the Covid-19 pandemic.

## Discussion

The article described some approaches to the implementation of adaptive technologies and students’ opinions, their reflections and approach to these methods and technologies.

Adaptive technologies have advantages and disadvantages. Among the advantages are productive learning and accurate recommendations for every student. The disadvantage is that they often require much more time for implementation compared to traditional learning and adaptive technologies do not always solve the problem of knowledge usage in ‘real’ life (Morze, Varchenko-Trotsenko, Terletska & Smyrnova-Trybulska, 2021).

The authors from Slovakia in their study “suggested methodology for creating personalised e-course adjusts the study content based on characteristics of each student stated by their initial knowledge, learning style, and motivation.” (Turcani & Mudrak, 2020, p. 573). We confirm the decision of the researchers that “one of the most commonly used LMS for the realization of personalised learning is Moodle, which after careful analysis was chosen in our case for experimental purposes” (pp. 580–582) and we used the Moodle system in our research as well.

We agree with the experts that “one of the essential advantages of using an innovative method for creating customized courses is by adapting it to the extent that its application can create customized courses without programming additional software solutions” (Zlatkovic et al., 2020, p. 811). The important conclusion in research conducted by Zlatkovic et al., is “for the implementation of the model, the tutor does not have to possess advanced knowledge in the field of information systems and software engineering.” (p. 811). The interesting proposal which should be taken into account in a future similar study that “the solution enables the creation of an adaptive environment for e-learning through the application of the methodological approach of the subject teacher.” (p. 811).

To introduce adaptive learning or its components in the learning process of the universities, e.g. specialized solutions, technologies and instruments can be used. However, the Moodle LMS which is already implemented at many universities offers a wide range of options for a teacher to plan, design, develop and implement adaptivity in the process of an e-learning course at all levels - adaptive schedule, adaptive content, adaptive sequence, adaptive control and adaptive assessment (Morze, Varchenko-Trotsenko, Terletska & Smyrnova-Trybulska, 2021).

The aims of creating an adaptive educational system is to fully meet the need for individualization using the technologies of problem-based testing and cognitively interested navigation in the educational material by students (Pishvanova, 2015). The motivation, the expectations and the individual student’s preferences taking into account their e-learning courses using the adaptive learning elements could increase the effectiveness of learning and teaching. The comprehensive and deep pedagogical, psychological and technological aspects should be the foundational elements in such research.

Research shows no significant moderation by nationality, but does show that the majority of Polish students (67.8%) and the majority of Ukrainian students (78.5%) showed their preference for electronic training (e-learning courses) and this is interesting, as it is the main topic of this study. The students from both countries gave a positive assessment of their participation in e-learning courses, which are considered to be their preferred educational tools. One of the „striking characteristics of the Net Generation students is speed. When speaking about young people, we usually say, ‘They want it right now’. This means these people want to learn quickly …. They need quick access to training materials, and e-learning tools” (Morze, Smyrnova-Trybulska & Umryk, 2015, p.480).

Polish students significantly more often (61.0%) chose personalised study materials, as opposed to31.4% of Ukrainian students. Simultaneously, students from the Ukraine significantly more often choose MOOCs and they significantly more often chose applications. We could mention that „one of the most popular platform for MOOC is Coursera. This project tries to connect people to a great education so that anyone around the world can learn without limits.” (Morze, Smyrnova-Trybulska & Umryk, 2015, p.480) Coursera has a long tradition and has more than 1 million users around of the worlds. The Polish MOOC’s platform Navoica (www.navoica.pl) has only been online since 2018 and was actively developed after running the NCBR *(Narodowe Centrum Badań i Rozwoju (National Centre for Research and Development))* project „Direction to the MOOCs”, supported by European funds (*Program Operacyjny Wiedza Edukacja Rozwój (Operational Program Knowledge Education Development*)) (POWR.03.01.00-IP.08–00-MOC/18), in particular, „MOOCs for Sciences of Education” project, fulfilling at the University of Silesia, Faculty of Arts and Sciences of Education (2019–2021).

There is no significant difference between the answers on the question: *Possibility of choosing convenient, comfortable time for study* and *Availability of materials in various formats (lecture texts, presentations, text files, films, discussion forums, tests,* etc. and *Ability to perform online / offline tasks and send the results of their implementation to the course.*

Additional comments and suggestions for improving the functionality of e-learning courses during the Covid-19 pandemic were defined in question 5: *Please rate the usefulness and effectiveness of the tools and websites used during the distance learning requirement of the Covid-19 pandemic on a scale of 1–5 (1 = the lowest grade, 5 = the highest grade).* The majority of both groups of students agree and obtained for the answer a median (4.0): Electronic training (e-learning courses); Personalised study materials; Video channels; Social media; Required material search engines.

The intercultural Aspects of Higher Education in particular in Ukraine was described in Korobochka et al. (2014). The authors stressed that “international cooperation and the success of cross-cultural communication is often based on personal relationships and, therefore, they are dependent on the individual communicative abilities of the partners” Korobochka et al. 2014, p.59). The conceptual aspects: law analysis, the ethical, human, technical, social factors of Development ICT, e-learning and intercultural development in different countries setting out the previous new theoretical model and preliminary findings was described in (Kommers et al., 2015).

The US and BGKU have a long tradition of cooperation in the framework of different activities and projects, e.g. an IRNet poject (7FP, Marie Curie Action, 2014–2017, www.irnet.us.edu.pl), Erasmus+ mobility programme, joint organizing of the DLCC conference (www.dlcc.us.edu.pl), joint MA programme prepared “E-learning management in cultural diversity” (Smyrnova-Trybulska, Morze, 2019) and others. We plan to continue our relationship on the different level and by strengthening our cooperation, research and collaboration, develop an innovative e-environment, implement more advanced ICT tools, methods and components of adaptive learning, in particular in the Moodle course, to achieve the highest possible effectiveness of the learning and teaching process.

### Future lines of research

Considering the above results, it is necessary to elaborate on and implement the specific training methodology to introduce the elements of personalised learning in the basic academic training of future teachers and postgraduates. This training should concentrate, first and foremost, on adaptive learning as the core aims and content for adequate and competent development. If this recommendation is implemented, future teachers will be able to teach in a more interactive, motivating and personalized way by applying the elements of adaptive and personalised learning. This system will take into account the students’ individual abilities, predispositions and preferences, as well as the administration’s and parents’ expectations for the effectiveness and high quality of learning in these trying times.

### Limitation

Within the context of the limitations of the analysis of the study, it would be interesting to expand the research sample, as well as to be able to submit a pretest and postest questionnaire to students to analyze if there are differences in their perceptions before and after the implementation of the elements of adaptive learning in the Moodle e-course. As a future direction of study, the authors are interested in expanding the research questions, along with the perception of the effectivness of the researched adaptive learning elements, their impact on motivation and the quality of learning and teaching. That is why the focus will be on proposing an experimental study (with a control group) in which we can analyze both the grades in the same academic course with several groups, in which personalised learning using the Moodle LMS system and other similar systems might shed light on a more timely methodology, as well as show the motivation of students with respect to the educational process.

## Conclusions

In the centre of the adaptive learning design process, there is a mentor, tutor or teacher who can choose the ways of implementing the expectations of students and their educational needs and learning styles. Furthermore, because one of the main tasks in the implementation of personalization and adaptive learning is the preparing of training courses for lectures, MOOCs and their implementation, the role of activities is no more limited to formal didactic contents, for instance the presenting and control of testing, but also requires the design of practical and creative tasks, project supervision and support from instructors and students learning basic time management skills. This could be in the form of micro-courses / microlearning, online training, e-learning courses, MOOC, tutoring, mentoring and the examination of case studies.

As shown in this article, the adaptive options are available in the setup of the Moodle system. The best examples are components such as Lesson or Quiz that expound upon the creation of students’ knowledge and evaluating said knowledge. If they attain a certain score, say 50%, and above, they go on to the next part of the lesson in the course framework or an available new set of activities (content, tests). If they get a lower score, they continue to a set of earlier content that will allow them to review the materials and check a portion of their knowledge. They have to complete these and then, either retake the original quiz or take a new one (their preference), this practice allows them to go on to the next part of the materials if they get the score the instructor requires. The teacher decides upon the requirements for the completion and qualifying results of the course. Other activities can be set up so that the student is required to view them to complete the assigned task; moreover, it is possible to have students check off the activity by themselves, however, in this case, the instructor will have to administer a test or quiz to verify that the student has internalized the required knowledge. The content for students may vary, for example, depending on the level of knowledge shown in the pre-test, learning style, and student preferences. The presented study in the two countries context proved the expectation of students from both countries. In both Polish and Ukrainian universities, there are technical, organizational and personnel conditions that will allow for a fairly quick implementation of the described concept and for the next stage of research with the analysis of students’ opinions concerning the results of the changes introduced in the courses.

## Data Availability

The datasets generated during and analysed during the current study are available from the corresponding author on reasonable request. During the study, an online anonymous survey in questionnaire form was administered to 59 students at the University of Silesia in Katowice, Poland and 121 students at the Borys Grinchenko Kyiv University, Ukraine between March–June 2020. Questionnaire in Polish and questionnaire in Ukraine were available on Google Form online. After finishing of the Research the Questionnaire they were closed. In case will need of datas for evaluators they could be available by corresponding author.

## Appendix 1

The survey content:

Q1. What digital educational tools do you prefer? *

1. Electronic training (e-learning courses)
2. Massive Open Online Courses (MOOCs)
3. Personalized study materials
4. Video channels
5. Mobile applications
6. Social networks
7. Search engines for the necessary materials
8. Audio books (audiobooks)
9. E-books (ibooks)
10. Other:

Q2. The University of Silesia/Borys Grinchenko, Kyiv University uses the e-learning system, please indicate the positive features and conditions of using e-learning courses:

1. Possibility to choose a convenient, comfortable time for study
2. Possibility of completing the course tasks and sending them to be checked gradually in turn
3. Possibility of returning to the course materials and individual topics several times
4. Availability of materials in various formats (lecture texts, presentations, text files, films, discussion forums, tests, etc.)
5. Ability to perform online/offline tasks and send the results of their implementation to the course instructor
6. Other:

Q3. Please indicate any shortcomings in the use of e-learning courses:

1. Teaching materials do not take into account the specificity of perception of information by students
2. Limited training time within the course
3. Lack of personalization in the learning process
4. Test homogeneity
5. Other:

Q4. Please indicate what activities can improve the quality of e-learning courses:

1. Choosing the order of study materials and performed tasks
2. Determining the scope of differentiating the knowledge provided by the course (basic, standard, advanced)
3. Choosing the learning dates from the different topics of the training materials
4. Choosing the format for the delivery/presentation of educational materials
5. Choosing the level of the complexity of tasks and tests
6. Other:

Additional comments and suggestions for improving the functionality of e-learning courses.

Q5. Please rate the usefulness and effectiveness of the tools and websites used during the distance learning requirement of the Covid-19 quarantine pandemic on a scale of 1–5 (1 = the lowest grade, 5 = the highest grade):

1. Electronic training (e-learning courses)
2. Massive Open Online Courses (MOOCs)
3. Personalized study materials
4. Video channels
5. Mobile applications
6. Social media
7. Required material search engines
8. Audio books (audiobooks)
9. E-books (ibooks)
10. Other

## Declarations

### Additional comments

Some of the survey results at the Borys Grinchenko Kyiv University BGKU were presented at the IConMaSTEd conference in Kryvyi Rih on 15–17 October 2020 and was published in the conference paper “Implementation of adaptive learning at higher education institutions by means of Moodle LMS”.

The current article includes a more comprehensive description of the theoretical background of adaptive learning and a more deep comprehensive statistical analysis of the comparison between the University of Silesia in Katowice and at the Borys Grinchenko Kyiv University, as well as additional and extended methodology, results, discussion, and conclusions. This article is only partially overlap (up to 10–20%) with Morze, N., Varchenko-Trotsenko, L., Terletska, T. & Smyrnova-Trybulska, E (2021). “Implementation of adaptive learning at higher education institutions by means of Moodle LMS”. Journal of Physics: Conference Series 1840 (2021) 012062 IOP Publishing doi:10.1088/1742-6596/1840/1/012062
